# Electrochemical Engineering of Emerging 2D Materials beyond Graphene: Progress and Prospects

**DOI:** 10.1002/smtd.202500197

**Published:** 2025-07-11

**Authors:** Dongdong Zhu, Lei Zhang, Li Wang, Huai Qin Fu, Zhenzhen Wu, Mengyang Dong, Yu Zou, Yaojie Lei, Xuan Zhang, Yu Lin Zhong, Liang Wang

**Affiliations:** ^1^ Jiangsu Key Laboratory of Atmospheric Environment Monitoring and Pollution Control Collaborative Innovation Center of Atmospheric Environment and Equipment Technology School of Environmental Science and Engineering Nanjing University of Information Science and Technology Nanjing 210044 China; ^2^ Centre for Catalysis and Clean Energy School of Environment and Science Griffith University Gold Coast Queensland 4222 Australia; ^3^ College of Materials Science and Engineering Donghua University Shanghai 201620 China; ^4^ Centre for Clean Energy Technology School of Mathematical and Physical Sciences University of Technology Sydney Sydney NSW 2007 Australia; ^5^ Queensland Micro‐ and Nanotechnology Centre School of Environment and Science Griffith University Nathan Queensland 4111 Australia

**Keywords:** electro‐deposition, electro‐etching, electro‐exfoliation, electronic device, electro‐topotactic‐transformation, energy conversion and storage, graphene‐like 2d materials

## Abstract

Following the remarkable success of graphene, graphene‐like 2D materials have garnered significant attention over the past decade due to their extraordinary physical and chemical properties. Despite the high demand for these materials in industry, developing facile, and cost‐effective methods for large‐scale production and functionalization under ambient conditions remains a challenge. As innovative green growth and modification strategies, electrochemical engineering techniques provide economic advantages in scalable manufacturing and surface manipulation. In this review, the established electrochemical techniques including intercalation‐exfoliation, deposition, etching, and topotactic transformation are summarized for producing a diverse range of emerging 2D materials and precisely tailoring their surface functional groups. Furthermore, numerous applications of these engineered 2D nanomaterials in energy and electronic fields, such as batteries, electrocatalysis, electronics, and optoelectronics are highlighted. Finally, it is concluded by outlining the remaining challenges and offering these perspectives on future research directions in this burgeoning field of advanced manufacturing.

## Graphical abstract

Graphene‐like 2D materials have recently attracted significant attention due to their exceptional physical and chemical properties. This review summarizes the established electrochemical engineering techniques applied to produce a diverse range of emerging 2D materials and precisely tailor their surface functional groups. Additionally, it highlights numerous applications of these engineered 2D materials in the energy and electronics fields.

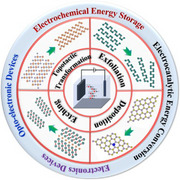

## 1 Introduction

After the discovery of graphene with superior properties in 2004, emerging graphene‐like 2D materials, such as elemental 2D materials, transition metal dichalcogenides (TMDs), MXenes, are attracting huge attention from the scientific community and commercial enterprises, which are playing an increasingly significant role in industrially essential applications in catalysis, energy storage, optoelectronic, et al.^[^ ^1^, ^2^, ^3^, ^4^, ^5^ ^]^ 2D materials often exist in bulk form as vertical stacks of layers with strong in‐plane covalent bonds and weak interlayer bonding, allowing the exfoliation of bulk precursors into atomically thin nanosheets.^[^ ^6^, ^7^ ^]^ The structural and electronic properties of monolayer or few‐layer 2D materials often differ remarkably from those of their 3D counterparts.^[^ ^8^, ^9^ ^]^ A common feature among 2D materials is their thickness‐dependent electronic structure.^[^ ^6^ ^]^ For instance, some 2D semiconducting materials exhibit an indirect‐to‐direct band gap transition as they are thinned down to monolayers, which can facilitate better performance in electronic/optoelectronic applications, such as photoluminescence.^[^ ^9^ ^]^ Additionally, the activated surface area and electronic conductivity can be significantly enhanced with reducing the vertical dimensionality in 2D materials, resulting in a higher density of active sites, which will improve the efficiency of energy conversion and storge, like batteries and electrocatalysis.^[^ ^2^, ^4^ ^]^ In contrast, 3D bulk materials often suffer from limited active sites and poor conductivity, which restrict their potential in these advanced applications.^[^ ^3^, ^6^ ^]^ Significant efforts have been devoted to preparing 2D atomic layers via different synthetic approaches, including mechanical exfoliation, molecular beam epitaxy, chemical vapor deposition, and chemical or liquid‐phase exfoliation.^[^ ^10^, ^11^, ^12^ ^]^ These fabricating methods often require special or harsh conditions, such as high temperature, high vacuum, high‐cost specialized instruments.^[^ ^13^, ^14^ ^]^ Due to these limits, these functional 2D materials have not been produced in large volumes at ambient conditions, which hinders them from commercialization.^[^ ^15^, ^16^ ^]^

Electrochemical engineering, one of the most active areas of knowledge, has contributed significantly to industrial development by manufacturing chemicals and functional materials.^[^ ^17^, ^18^ ^]^ In recent years, electrochemical engineering has been widely explored and practized to produce monolayer or few‐layer graphene via the exfoliation approach.^[^ ^19^, ^20^, ^21^ ^]^ Moreover, fast and efficient surface modification of graphene nanolayers can also be achieved by in situ electrochemical routes with the introduction of diazonium salts, which can controllably tune the electronic properties of graphene.^[^ ^22^, ^23^ ^]^ Due to the unique advantages of low cost and easy implementation, electrochemical engineering approaches have become promising strategies for fabricating ultrathin 2D materials in large quantities.^[^ ^24^ ^]^ These electrochemical engineering routes can be conducted at ambient conditions, and effectively reduce or even eliminate the generation of chemical wastes by recycling electrolytes, although these technologies are still in the early stage.^[^ ^25^ ^]^ Previous review articles on electrochemical engineering of graphene‐like 2D materials are only centered on the electrochemical exfoliation approach.^[^ ^26^, ^27^, ^28^, ^29^ ^]^ However, some other significant electrochemical engineering strategies (e.g., etching, deposition, and topotactic‐transformation) are usually not covered. Therefore, a timely and comprehensive review on the development of electrochemical processing techniques for the synthesis and surface modification of 2D nanostructures is essential and meaningful to facilitate further research in this area.

**Figure 1:**
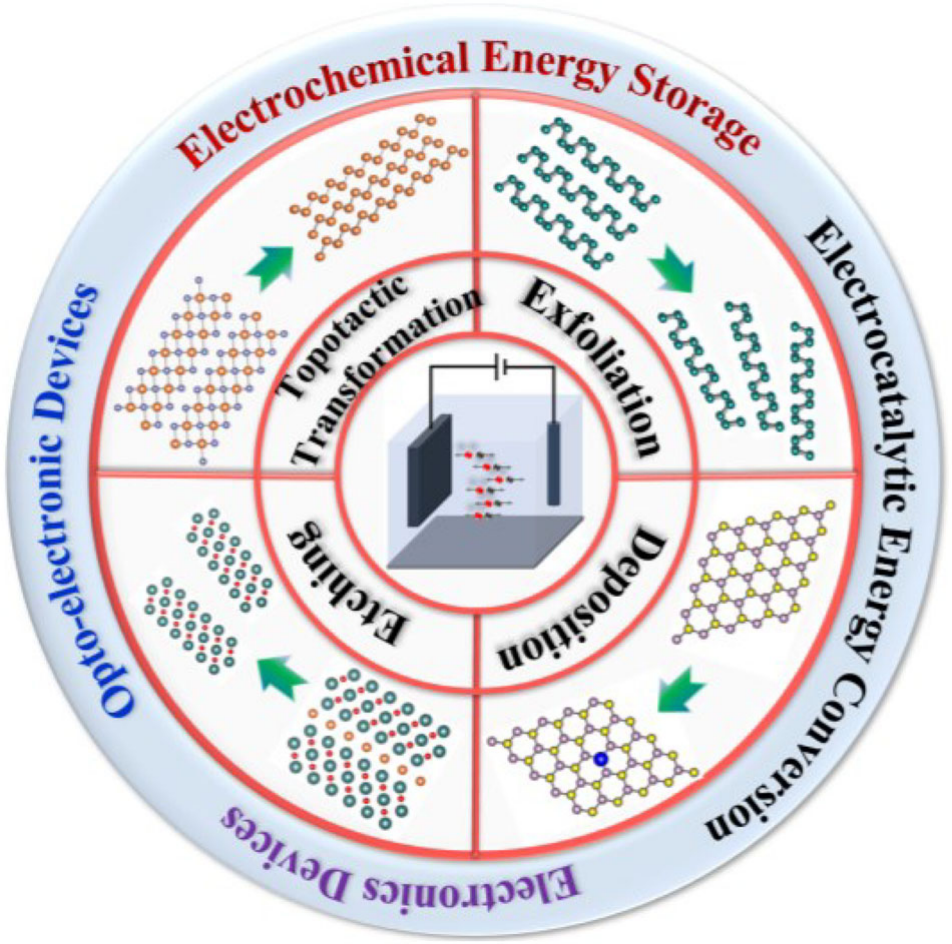
An overview diagram showing electrochemical engineering strategies of emerging 2D materials and their promising applications.

**Figure 2:**
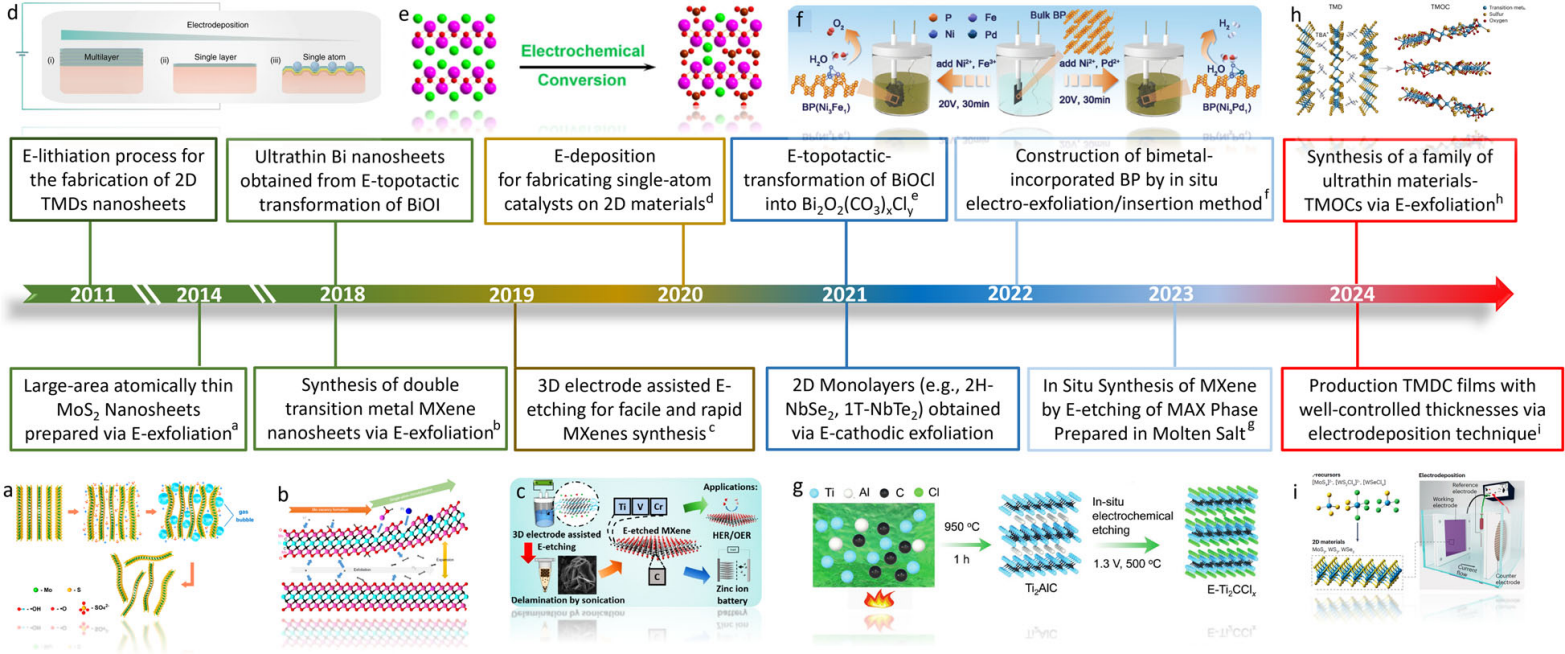
Milestones in development progress of electrochemical engineering techniques.^[^ ^34^, ^35^, ^36^, ^37^, ^38^, ^39^, ^40^, ^41^, ^42^, ^43^, ^44^, ^45^, ^46^ ^]^ Reproduced with permission.^[^ ^34^ ^]^ Copyright 2020, Springer Nature. Reproduced with permission.^[^ ^35^ ^]^ Copyright 2022, Springer Nature. Reproduced with permission.^[^ ^36^ ^]^ Copyright 2014, American Chemical Society. Reproduced with permission.^[^ ^37^ ^]^ Copyright 2019, American Chemical Society. Reproduced with permission.^[^ ^38^ ^]^ Copyright 2018, Springer Nature. Reproduced with permission.^[^ ^39^ ^]^ Copyright 2018, Springer Nature. Reproduced with permission.^[^ ^40^ ^]^ Copyright 2024, Chinese Chemical Society. Reproduced with permission.^[^ ^41^ ^]^ Copyright 2024, Royal Society of Chemistry. Reproduced with permission.^[^ ^42^ ^]^ Copyright 2022, Springer Nature. Reproduced with permission.^[^ ^43^ ^]^ Copyright 2018, Springer Nature. Reproduced with permission.^[^ ^44^ ^]^ Copyright 2022, Wiley‐VCH. Reproduced with permission.^[^ ^45^ ^]^ Copyright 2025, Springer Nature. Reproduced with permission.^[^ ^46^ ^]^ Copyright 2025, Springer Nature.

In this review, we provide a comprehensive discussion on the recent advances in the field of electrochemical engineering for the fabrication and functionalization of emerging 2D materials beyond graphene (**Figure** **1**). Electrochemical engineering approaches, including exfoliation, etching, deposition, and topotactic‐transformation, can offer a means of manufacturing 2D atomically thin nanosheets, and the as‐synthesized ultrathin 2D materials can be further electrochemically functionalized to achieve desired properties, such as fast charge transport mobility, modified electronic structure, high concentrations of active sites, etc.^[^ ^30^, ^31^, ^32^, ^33^ ^]^ **Figure** **2** displays the key progress in the development of electrochemical engineering of 2D materials.^[^ ^34^, ^35^, ^36^, ^37^, ^38^, ^39^, ^40^, ^41^, ^42^, ^43^, ^44^, ^45^, ^46^ ^]^ Therefore, electrochemical engineering approaches will make a great contribution to the development and growth of next‐generation 2D functional nanomaterials or nano‐heterostructures for potential applications.

## 2 Electrochemical Engineering Mechanism

Electrochemical engineering strategies can not only be performed to fabricate atomically thin 2D nanostructures but also be applied to controllably introduce functional groups or sites onto surfaces of 2D nanolayers and precisely tailor the coordination environments by controlling electrochemical parameters (e.g., the current density and applied voltage) and reaction conditions (e.g., pH value, electrolyte, the solvent concentration, types of solute metal atoms, and external heating and/or pressure).^[^ ^47^, ^48^, ^49^, ^50^ ^]^ At present, four electrochemical engineering strategies including electrochemical exfoliation, etching, reduction, and deposition have been reported for the preparation of ultrathin 2D materials. The definition of electrochemical exfoliation has remained ambiguous. In some studies, electrochemical etching is also referred to as electrochemical exfoliation, as the extraction of metal ions during etching can lead to the exfoliation of layers. In this review, however, electrochemical exfoliation is specifically defined as an electrochemical process involving the intercalation of molecules and the expansion of 2D layered materials, without typical redox reactions occurring. Conversely, electro‐topotactic transformation refers to the electrochemical anion exchange reaction or the reduction of precursors for synthesizing metal‐based materials. During this transformation process, exfoliation can occur simultaneously, as anion exchange or anion oxidation weakens the bonding between structural layers. Thus, in the synthesis of 2D materials, electro‐etching and electro‐topotactic transformation techniques sometimes act synergistically in facilitating exfoliation.

### 2.1 Electro‐Exfoliation

**Figure 3:**
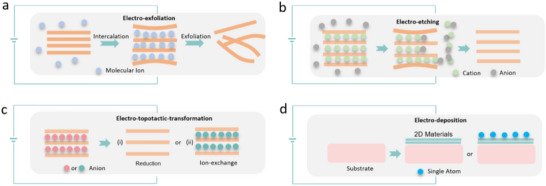
Schematic illustration of electrochemical engineering processes for preparation and modification of emerging 2D materials: a) electro‐exfoliation, b) electro‐etching, c) electro‐topotactic‐transformation, and d) electro‐deposition. Reproduced with permission.^[^ ^34^ ^]^ Copyright 2020, Springer Nature.

Employing electric flow in solutions to facilitate the intercalation of guest molecules or ions can be an effective method for exfoliating 3D layered crystals into individual flakes. Electro‐exfoliation approaches can take advantage of the conductive properties of bulk precursors to intercalate molecules or ions between the interlayer gaps (**Figure** **3**a).^[^ ^51^, ^52^ ^]^ In the electrochemical exfoliation process, the organic molecules (e.g., quaternary ammonium cation and Lewis acid with tunable molecular size from 10 to 20 Å) are usually placed as the intercalants to break the van der Waals interactions between layers for the successful production of high‐quality 2D nanosheets, such as TMDs materials (e.g., MoS_2_, MoTe_2_, TaS_2_, and VSe_2_,), elemental 2D materials (e.g., black phosphorous (BP), antimony, silicene, and arsenene), and other 2D layered materials (e.g., Mxene, metal‐organic frameworks (MOFs), and In_2_Se_3_).^[^ ^28^, ^53^ ^]^

### 2.2 Electro‐Etching

Chemical etching involving multi‐electron transfer processes is intrinsically an electrochemical process, often raising considerable safety and environmental concerns.^[^ ^54^ ^]^ Low‐cost and single‐step electrochemical etching techniques can be an efficient alternative strategy to minimize these issues by using electrochemical redox reactions, which can be utilized to fabricate high‐quality 2D nanolayers via processing bulk precursors (Figure 3b).^[^ ^55^ ^]^ In typical electrochemical etching process, one or more elements can be selectively extracted from the precursor materials to generate 2D layered nanostructures.^[^ ^56^ ^]^

### 2.3 Electro‐Topotactic‐Transformation

Topotactic transformations between related layered structures represent a potent emerging method for synthesizing 2D materials, involving the partial removal, insertion, or exchange of anions. Electro‐topotactic‐transformation is another highly effective method for the fabrication of 2D materials from native materials (Figure 3c).^[^ ^57^ ^]^ Under electrochemical reduction potentials, 2D metal‐based precursors undergo structural rearrangements, resulting in their partial or full reduction to metal states via direct electron transfer from the working electrode, or their partial or full transformation into new 2D materials through anion exchange.^[^ ^58^ ^]^

### 2.4 Electro‐Deposition

As an inherently solution‐based process where material growth occurs directly on the electrode surface, electrodeposition is a highly promising method due to its scalability and ability to selectively grow in specific areas. The electrochemical deposition approach has been widely used for polymer coatings and the construction of nanoporous architectures (Figure 3d).^[^ ^59^, ^60^ ^]^ For ultrathin 2D materials, the highly electronegative surface atoms can act as the accessible attacking sites for ligands, enabling them to be potential substrates for the confinement of various single atoms or epitaxial growth of other 2D layered compounds.^[^ ^61^, ^62^ ^]^ Electrodeposition can be potentially utilized to create active dopant sites onto 2D supports or build epitaxially 2D heterostructures.^[^ ^50^ ^]^ The electrochemical deposition takes place at the electrode‐electrolyte interface, in which the product can be effectively deposited on the surface of the working electrode in the form of few‐layer films or single atoms.^[^ ^63^ ^]^

Conventional synthesis methods for 2D materials are often time‐intensive, requiring prolonged agitation or sonication, sometimes lasting hours or even days. Additionally, many of these approaches involve harsh conditions, such as high temperatures (>100 °C), high vacuum, and hazardous chemicals—including strong acids, bases, or toxic organic solvents—necessitating extensive post‐synthesis washing and separation steps for purification. In contrast, electrochemical engineering strategies provide a more controlled and efficient route for synthesizing novel 2D nanostructures under ambitious conditions. These processes are not only simpler but also highly scalable, offering a promising alternative for 2D material fabrication. A significant limitation of these electrochemical engineering strategies is that the electrochemical processing necessitates bulk crystals to be electrically conductive, meaning the crystals must be metallic, semi‐metallic, or semiconducting. Consequently, it is inherently challenging to apply these strategies to insulating 2D layered materials. Furthermore, the properties of the bulk crystal used as the electrode, including the dimensions, morphology, and mechanical characteristics, significantly affect the productivity and electronic properties of the as prepared 2D materials

## 3 Electrochemical Engineering Strategies

### 3.1 Electro‐Exfoliation for Fabricating 2D Materials

Electrochemical exfoliation of 2D bulk materials has emerged as an efficient and quick way to generate atomically thin nanolayers such as elemental 2D materials and TMDs, in large quantity, which is among the most widely investigated electrochemical engineering strategies thus far.^[^ ^27^ ^]^

#### 3.1.1 Elemental 2D Materials

**Figure 4:**
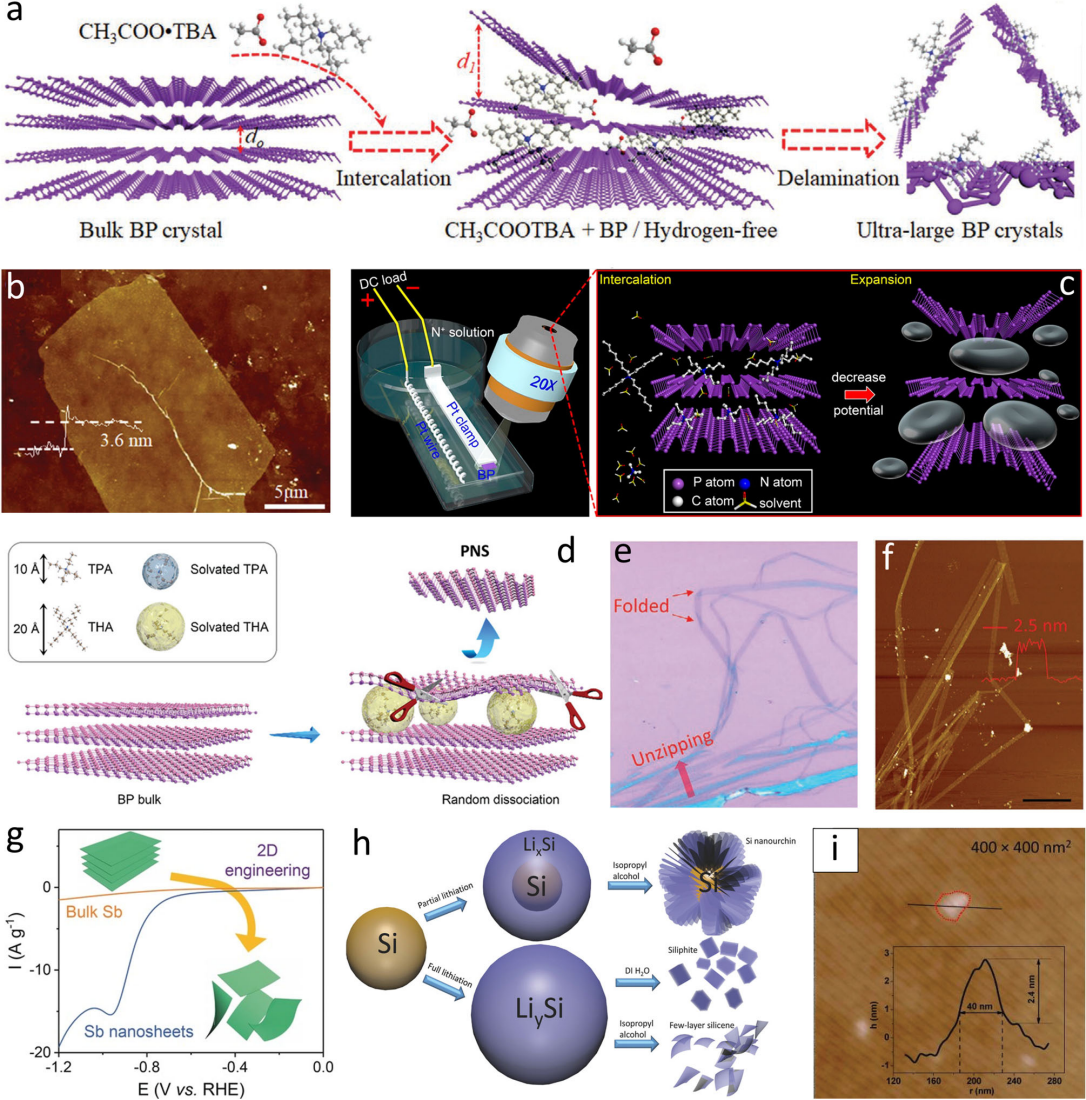
Electrochemical exfoliation of elemental 2D materials. a) Schematic illustration of the hydrogen‐free electrochemical delamination of ultra‐large few‐layer BP. b) AFM image of ultrathin BP layers. Reproduced with permission.^[^ ^69^ ^]^ Copyright 2021, Wiley‐VCH. c) Schematic illustration of the ultrafast electrochemical intercalation and expansion of bulk BP toward high‐yield fabrication of phosphorene. Reproduced with permission.^[^ ^70^ ^]^ Copyright 2018, American Chemical Society. d) Proposed electrochemical exfoliation mechanism of phosphorene nanoribbons. e) An optical microscopy image and f) AFM topography image of phosphorene nanoribbon. Reproduced with permission.^[^ ^72^ ^]^ Copyright 2021, Wiley‐VCH. g) Schematic illustration of the electrochemical exfoliation procedure for preparation of few‐layer Sb nanosheets. Reproduced with permission.^[^ ^74^ ^]^ Copyright 2017, Wiley‐VCH. h) Schematic of the electrochemical lithiation and delithiation process of silicon for fabrication of silicene. i) AFM image of exfoliated silicene nanolayers. Reproduced with permission.^[^ ^75^ ^]^ Copyright 2018, Wiley‐VCH.

The electrochemical exfoliation technique has become a vital tool in the development of elemental 2D materials designed for specific requirements, which can also offer a means of controllable functionalization at the molecule level by controlling electrochemical parameters (**Table** **1**).^[^ ^2^, ^64^ ^]^ As one of the typical elemental 2D materials, BP exhibits various remarkable properties depending on atomic and electronic structures.^[^ ^65^, ^66^ ^]^ Traditional sonication‐assisted liquid‐phase exfoliation method often generates irregularly shaped few‐layers BP, because P‐P bonds can be broken randomly.^[^ ^28^, ^67^ ^]^ The electrochemical exfoliation approach enables the in situ production of few‐layer BP nanosheets with ultra‐large domain size.^[^ ^68^ ^]^ In a typical two‐electrode electrochemical system with bulk BP crystal as a cathode, a Pt sheet as the anode, and the weak Lewis acid tetra‐n‐butylammonium acetate (CH_3_COOTBA) as the intercalation molecule, the anion (i.e., CH_3_COO^−^) can effectively penetrate the inner layers of bulk BP under an external voltage, breaking the van der Waals force between the inner layers (**Figure** **4**a).^[^ ^69^ ^]^ Due to the weak interaction between cations (TBA^+^) and coordination anions (CH_3_COO^−^) in the weak Lewis acid, the hydrogen evolution reaction during electrochemical exfoliation process is significantly inhibited. Benefiting from the advantages of the weak Lewis acid delamination and a hydrogen‐free electrochemical reaction, the mechanical damage of P‐P bonds in the crystal can be effectively avoided (Figure 4b). In the electrochemical exfoliation process of 2D materials, the size of solvated cations in electrolytes plays a critical role in modulating the intercalation and exfoliation efficiency. Using a nonaqueous solution consisting of tetraalkylammonium (TAA) salts and dimethyl sulfoxide (DMSO) as the organic electrolyte, an ultrafast electrochemical cathodic exfoliation of bulk BP can be achieved within minutes for scalable fabrication of high‐quality few‐layer BP with ultrahigh yield (>80%) (Figure 4c).^[^ ^70^ ^]^ In comparison with 2D nanolayers, ultrathin nanoribbons with 2D structures possess more edge active sites for catalytic reactions.^[^ ^71^ ^]^ A facile electrochemical cathodic exfoliation was exploited for generating zigzag‐edged phosphorene nanoribbons in high yield (>80%) using a dual‐electrode electrochemical setup with quaternary ammonium cations (tetrapropylammonium or tetrahexylammonium (THA)) as intercalants and the propylene carbon as the electrolyte (Figure 4d).^[^ ^72^ ^]^ With the electrochemical intercalation of organic molecules into bulk BP, the generated internal strain could induce the dissociation of P‐P bonds along zigzag direction, enabling the exfoliation of phosphorene nanoribbons (Figure 4e). The exfoliated phosphorene nanoribbon has a thickness of 2.5 nm confirmed through AFM observation (Figure 4f). Apart from BP, atomic layers of other elemental 2D materials (e.g., Sb and As) can also be facilely prepared by the electrochemical cathodic exfoliation of initial bulk crystals (Figure 4g).^[^ ^73^, ^74^ ^]^ In addition, the electrochemical lithiation and delithiation process was also demonstrated for the exfoliation of elemental 2D materials, such as silicene.^[^ ^75^ ^]^ In a typical lithiation process, the interaction of lithium ions into Si‐based electrodes can be performed in Li‐ion coin cells during discharging (Figure 4h). Then the subsequent delithiation can be carried out by rinsing the obtained electrodes with protic solvents (e.g., water and isopropyl alcohol) in an ultrasonication bath, leading to the expansion and exfoliation of bulk Si (Figure 4i).

**Table 1:** Electrochemical exfoliation of 2D elemental materials.

Product	Precursor	Electrolyte	Electrochemical engineering parameters	Dimensions (Thickness, Size)	Refs.
Holey Phosphorene	BP Crystal	1 M H_2_SO_4_	3 V or 4 V (Two‐Electrode System)	≈1.5 nm, 300–900 nm (Pore Size 55.08 ± 4 nm)	[21]
Phosphorene	Bulk BP Crystals	0.5 M H_2_SO_4_	+3 V for 2 h (Two‐Electrode System)	**/**	[47]
Ultra‐large BP Nanosheets	Bulk BP Crystal	2 mM Weak Lewis Acid (CH_3_COOTBA)	20 V for 30 min (Two‐Electrode System)	4.1 nm, 77.6 ± 15.0 µm	[69]
Phosphorene	Bulk BP Crystal	0.01 M TAA (DMSO)	−5 V for 10 min	1.1–3.7 nm (Thickness)	[70]
Zigzag‐Phosphorene Nanobelts	Bulk BP Crystal	[BMIM]BF_4_/Distilled Water (Weight Ratio 1/2)	≈0.1 to ≈0.5 A cm^−2^ for 30 min (Two‐Electrode System)	≈2.7 ± 1.7 nm (Thickness)	[71]
2D Arsenene	Bulk As Crystal	0.01 M NH_4_PF_6_/DMF	−3.8 V vs Ag/ Ag^+^/AgNO_3_ for 5 h	≈0.6 nm, ≈1.5 µm	[73]
2D Sb Nanosheets	Bulk Sb Crystal	0.5 M Na_2_SO_4_	10 V (Two‐Electrode System)	**/**	[74]
2D Silicene Nanosheets	Si Powders	**/**	0.1–0.25 V vs Lithium (Li‐O_2_ Batteries)	2–3 nm, 30–100 nm	[75]
2D Antimonene Nanosheets	Sb Rod	1 M Tetramethylammonium Hydroxide	−5 V (Two‐Electrode System)	≈2 nm (Thickness)	[122]

#### 3.1.2 TMDs Materials

**Figure 5:**
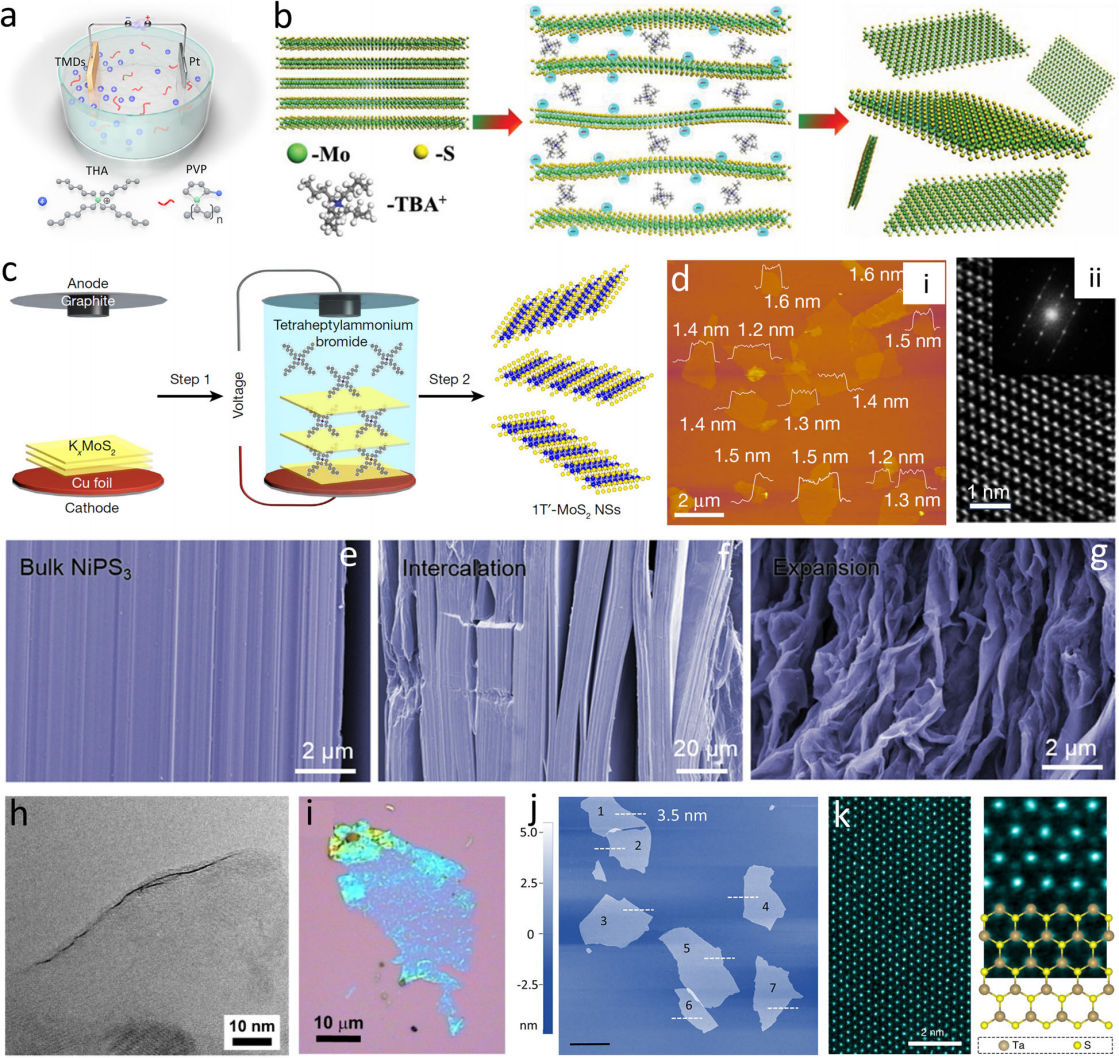
Electrochemical exfoliation of 2D TMDs materials. a) Schematic illustration of the two‐electrode electrochemical setup for the polymer‐assisted electrochemical exfoliation with THA as the intercalant and PVP as the surfactant. Reproduced with permission.^[^ ^76^ ^]^ Copyright 2021, American Chemical Society. b) Schematic representation of the possible mechanism of electrochemical exfoliation of MoS_2_ crystal. Reproduced with permission.^[^ ^77^ ^]^ Copyright 2019, Wiley‐VCH. c). Schematic representation of the synthesis of 1T′‐MoS_2_ nanosheets from bulk K_x_MoS_2_ crystals via electrochemical intercalation and exfoliation. d) Reproduced with permission.^[^ ^78^ ^]^ Copyright 2023, Springer Nature. Ex situ SEM images of NiPS_3_ electrochemically exfoliated for 0s e), 30s f), and 60s g) in tetra‐n‐butylammonium salts solution under a bias voltage of ‐3 V. Reproduced with permission.^[^ ^79^ ^]^ Copyright 2019, Wiley‐VCH. h) HRTEM and i) optical microscope images of single electrochemically exfoliated MoS_2_ monolayer. Reproduced with permission.^[^ ^36^ ^]^ Copyright 2014, American Chemical Society. j) AFM topography image of the electrochemically exfoliated 1T’‐MoTe_2_ nanolayers. Reproduced with permission.^[^ ^76^ ^]^ Copyright 2021, American Chemical Society. k) Atomic‐resolution STEM‐HAADF images of 2H‐TaS_2_ monolayer obtained by electrochemical exfoliation. Reproduced with permission.^[^ ^81^ ^]^ Copyright 2021, Springer Nature.

Apart from elemental 2D materials, electrochemical exfoliation can be an effective strategy to produce individual or few‐layer TMDs nanosheets (**Table** **2**).^[^ ^26^, ^29^ ^]^ A typical electrochemical exfoliation process of TMDs can be performed in a two‐electrode system with the Pt foil as the counter electrode, bulk TMDs materials as the cathode, and the quaternary ammonium cation, such as THA and TBAcation, as the intercalant (**Figure** **5**a).^[^ ^76^ ^]^ With a constant negative bias applied to the cathode electrode, the cations were driven to be intercalated into the interlamination of bulk TMDs crystals, significantly weakening the van der Waals interactions between layers and resulting in further lattice expansion. After the intercalation, a well‐dispersed ink solution consisting of 2D ultrathin nanoflakes can be obtained by low power ultrasonication or manual shaking (Figure 5b).^[^ ^77^ ^]^ The completion of the electrochemical exfoliation process can be achieved within minutes, much faster than liquid‐phase exfoliation methods. The electrochemical exfoliation mechanism of TMDs was investigated by linear sweep voltammetry, demonstrating that the THA cation was not able to insert into the host crystal of MoS_2_ at an initial low voltage and the cathodic current decreased with the expansion of MoS_2_ at more negative voltages. The crystal phase is a fundamental factor in shaping the electronic and structural properties of 2D TMDs, directly influencing their functions and potential applications. Electrochemical exfoliation has also emerged as an effective synthetic approach for producing 2D TMDs with distinct crystalline phases, such as 1T′‐MoS_2_.^[^ ^78^ ^]^ In this process, tetraheptylammonium bromide molecules were first intercalated into K_x_MoS_2_ crystals within an electrochemical cell (Figure 5c). Subsequent exfoliation yielded 1T′‐MoS_2_ nanosheets with an average thickness of 1.4 ± 0.4 nm and lateral dimensions reaching several micrometres, as characterized by AFM and atomic resolution HAADF‐STEM (Figure 5d). In addition, the morphological change of TMDs‐based electrode was also monitored by *ex‐situ* scanning electron microscopy (SEM), showing that closely stacked lamellar structures of bulk TMDs (Figure 5e) will evolve into accordion‐like structures (Figure 5f) and finally transform to roughened and wrinkled structures (Figure 5g).^[^ ^79^ ^]^ In addition, ultra‐large atomically thin MoS_2_ nanosheets with the lateral size of 50 µm can be fabricated by electrochemical exfoliation, which is much larger than those obtained by the mechanical, chemical, or liquid‐phase exfoliation methods (Figure 5h,i).^[^ ^36^ ^]^ Besides, a wide range of high‐quality monolayer TMDs, including MoTe_2_ (Figure 5j), Nb(Se/Te)_2_, Ta(S/Se)_2_ (Figure 5k), Ti(S/Se)_2_, PdTe_2_ were also successfully produced via electrochemical exfoliation using quaternary ammonium cations as a cathodic intercalant.^[^ ^80^, ^81^ ^]^

**Table 2:** Electrochemical exfoliation of 2D TMD materials.

Product	Precursor	Electrolyte	Electrochemical engineering parameters	Dimensions (Thickness, size)	Refs.
Large‐Area MoS_2_ Nanosheets	Bulk MoS_2_	0.5 M Na_2_SO_4_	+10 V for 0.5‐2 h (Two‐Electrode System)	0.9‐1.3 nm, 5–50 µm	[36]
ReS_2_ Monolayer Flakes	ReS_2_ Single Crystal	0.005 M Tetraheptylammonium or Tetrapropylammonium Chloride	−5 V (Two‐Electrode System)	≈0.8 nm, >20 µm	[53]
PdTe_2_ Nanoparticles	PdTe_2_ bulk	0.25 M TBATFB	−5 V (Two‐Electrode System)	50 ± 20 nm	[80]

#### 3.1.3 Other 2D Materials

Apart from TMDs and elemental 2D materials, the electrochemical exfoliation strategy was also explored to synthesize other 2D materials (**Table** **3**), such as MOFs and MXenes, In_2_Se_3_.^[^ ^39^, ^82^, ^83^ ^]^ For example, Huang et al. reported ultrathin 2D MOF nanosheets based on coordination bonds synthesized via selective pillar removal and in situ electrochemical exfoliation of initial pillared‐layer MOF.^[^ ^82^ ^]^ Moreover, the electro‐exfoliation approach was also developed for the preparation of double transition metal MXene (Mo_2_TiC_2_Tx) nanosheets. During the exfoliation process, abundant Mo vacancies were in situ generated, serving as active sites for the immobilization of Pt single atoms and significantly enhance the electrocatalytic hydrogen evolution reaction (HER) activity of the MXene.^[^ ^39^ ^]^

**Table 3:** Electrochemical exfoliation of additional 2D materials.

Product	Precursor	Electrolyte	Electrochemical engineering parameters	Dimensions (Thickness, size)	Refs.
2D In_2_Se_3_ Flakes	α‐In_2_Se_3_ Crystals	THABr (DMF, 0.1 M)	−1 V to ‐10 V for 30 min (Two‐Electrode System)	4.0 nm, 8.6 µm (Average)	[83]
2D InSe Nanosheets	InSe Crystal	0.01 M THAB	−3.2 V for 2 h (Two‐Electrode System)	≈1.7 nm, ≈2 to ≈0.5 µm	[123]
Mo_2_TiC_2_T_x_ MXene	Mo_2_TiC_2_T_x_ MXene Powder	0.5 M H_2_SO_4_	20 mV s^−1^, 0 to −0.53 V vs RHE	/	[39]

### 3.2 Electro‐Etching for Synthesis of MXenes

As an emerging class of 2D materials, MXenes are attracting increasing attention owing to their exceptional electronic, mechanical, and optical properties.^[^ ^84^ ^]^ MXene nanosheets are usually fabricated by selective etching of bulk MAX phase (M = an early transition metal, A = an element from group 13 and 14, and X = carbon, nitrogen, or boron) with acid/alkali etchants or via molten salt etching route under high temperature and pressure.^[^ ^85^ ^]^ The harsh synthetic conditions have hindered the extensive development of MXenes. As a facile and green fabrication process, the electrochemical etching route has demonstrated promising selective etching effectiveness on MAX precursors by tuning electrochemical parameters.

**Figure 6:**
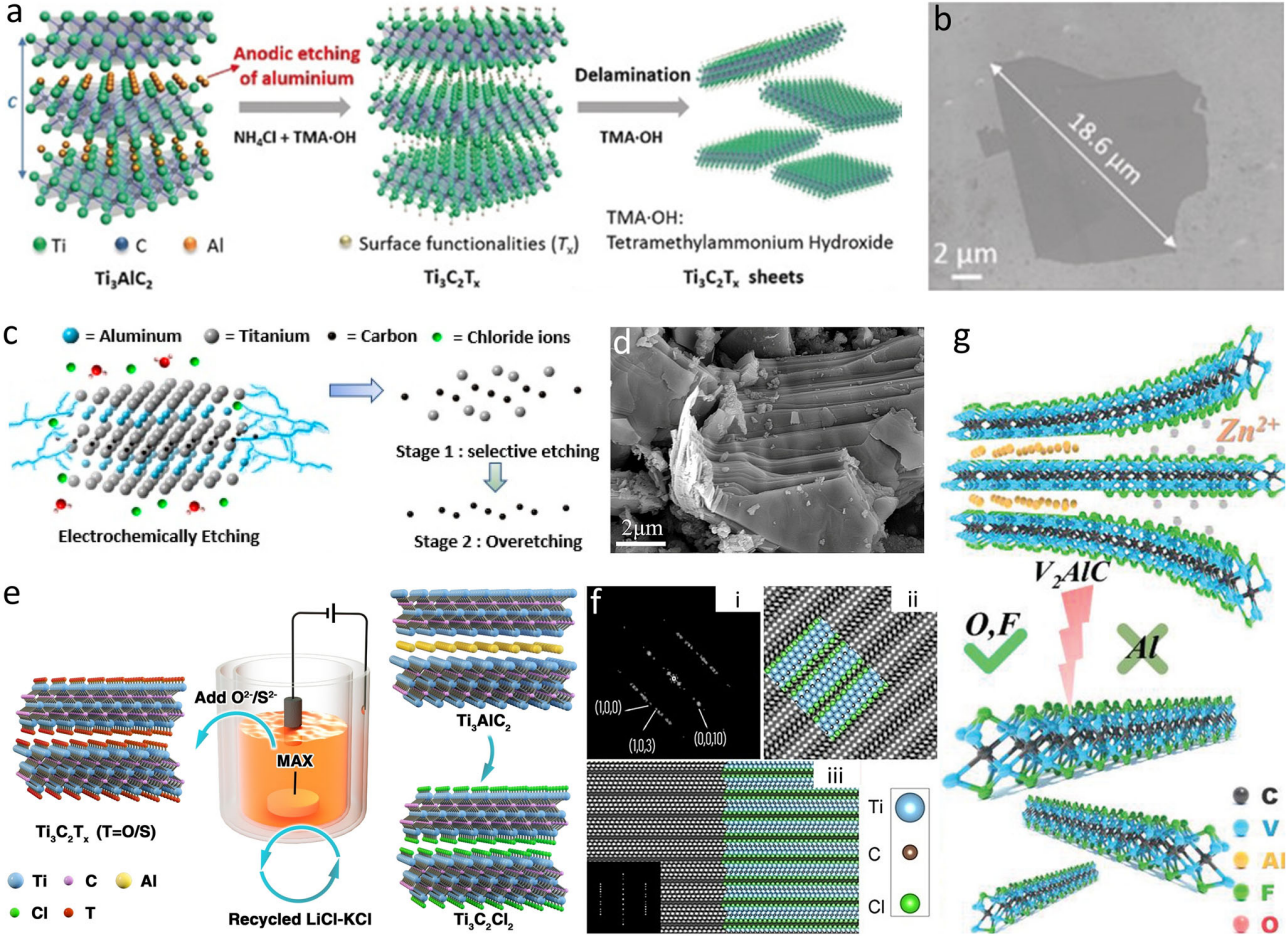
Electrochemical etching for fabrication of 2D materials. a) Schematic of the electrochemical anodic etching of bulk Ti_3_AlC_2_ in a binary aqueous electrolyte and the delamination of obtained Ti_3_C_2_T_x_. b) SEM image of single ultra‐large delaminated Ti_3_C_2_T_x_ nanosheet. Reproduced with permission.^[^ ^86^ ^]^ Copyright 2018, Wiley‐VCH. c) Proposed HF‐free electrochemical etching mechanism of MAX precursors in HCl electrolyte. d) SEM image of loosely packed Ti_3_C_2_T_x_ particles. Reproduced with permission.^[^ ^37^ ^]^ Copyright 2019, American Chemical Society. e) Synthesis of MXene with tunable surface terminations by molten‐salt‐assisted electrochemical etching of the MAX phase. f) Atomic‐resolution HAADF‐STEM image and the simulated atomic arrangement of Ti_3_C_2_Cl_2_. Reproduced with permission.^[^ ^88^ ^]^ Copyright 2021, Wiley‐VCH. g) Schematic illustration of the electrochemical etching mechanism for in situ synthesis of MXene in an aqueous zinc ion battery. Reproduced with permission.^[^ ^89^ ^]^ Copyright 2020, Wiley‐VCH.

Electrochemical etching has been employed as an effective strategy for the production of MXene nanolayers such as Ti_3_C_2_T_x_ (T = O, OH) by two‐electrode systems (**Figure** **6**a).^[^ ^37^, ^85^, ^86^, ^87^ ^]^ Due to fluoride‐free electrochemical etching, the obtained Ti_3_C_2_T_x_ (T = O, OH) nanoflakes do not possess any fluorine terminations (Figure 6b). Additionally, it was found that mild heating can accelerate the electrochemical etching process on precursor MAX materials with the low‐concentration hydrochloric acid as the etchant (Figure 6c).^[^ ^37^ ^]^ A thermal‐assisted electrochemical‐etching method was applied to prepare HF‐free MXenes (Ti_2_CT_x_, Cr_2_CT_x_, and V_2_CT_x_) with a 3D composite electrode which is composed of carbon black additive and carbon fiber cloth substrate (Figure 6d). Here the addition of carbon black and carbon fiber cloth can result in larger current densities during the electrochemical etching process. Moreover, a novel molten‐salt‐assisted electrochemical etching method has been designed and developed to synthesize fluorine‐free Ti_3_C_2_Cl_2_ (Figure 6e).^[^ ^88^ ^]^ The surface terminations of MXene can be in situ modified from Cl to O and/or S, which considerably shortens the modification steps and enriches the variety of surface terminations (Figure 6f). Besides, the electrochemical etching for the synthesis of 2D MXenes can be achieved during the charge and discharge processes in rechargeable batteries. For example, the precursor V_2_AlC can be in situ electrochemically etched to V‐based MXene inside the aqueous zinc ion battery using MAX as the cathode and F‐rich electrolyte as the etchant (Figure 6g).^[^ ^89^ ^]^ Additionally, electro‐etching has been explored for the preparation of other 2D materials, such as germanene.^[^ ^90^ ^]^ As the electro‐etching technique continues to advance, it is expected to facilitate the fabrication of a wider range of 2D materials (**Table** **4**).

**Table 4:** Electrochemical etching approach for producing atomically thin 2D materials.

Product	Precursor	Electrolyte	Electrochemical engineering parameters	Dimensions (Thickness, size)	Refs.
MXenes (Ti_2_CT*_x_*, Cr_2_CT*_x_*, and V_2_CT*_x_*)	MAX Phase Powder (Ti_2_AlC, V_2_AlC and Cr_2_AlC)	1 M HCl	0.3‐1 V vs RHE for 9 h	≈5 – 80 nm, >1 µm	[37]
Ti_2_CT_x_ MXene	Ti_2_AlC MAX	LiCl‐KCl Molten Salt	0.8 V (rGO‐Based) vs Quasi‐Reference Electrode for 24 h at 500 °C	≈6.5 nm (Thickness)	[44]
Ti_2_CT_x_ MXene	Bulk Ti_2_AlC	1‐2M HCl	+0.6 V vs Ag/AgCl for 1–5 days	**/**	[55]
Ti_3_C_2_T_x_ MXene	Bulk Ti_3_AlC_2_	1 M NH_4_Cl+ 0.2 M TMA·OH	+2.48 V vs SCE for 5 h	≈1.2 nm, 1 to 5 µm	[86]
Ti_3_C_2_T_x_ MXene	Bulk Ti_3_AlC_2_	10 M NH_4_F	1.30 and 19.49 mA cm^−2^ (Two‐Electrode System)	4 nm (Thickness)	[87]
Ti_3_C_2_Cl_2_ MXene	Ti_3_AlC_2_ Powder	LiCl‐KCl	0.365 V vs Ag/AgCl at 450 ^o^C	**/**	[88]
V_2_CT_X_ MXene	V_2_AlC MAX Powder	21 M LiTFSI + 1 M Zn(OTf)_2_	400‐600 cycles at 5 A g^−1^ (Zinc Ion Battery)	8.5 nm, >1 µm	[89]
Cl‐containing Ti_3_C_2_T_x_ MXene	Ti_3_AlC_2_ Blocks	0.8 M LiOH + 1.0 M LiCl	5.5 V for 5 h (Two‐Electrode System)	1.0 to 6.5 nm, 3.8 µm	[126]
2D Germanene	CaGe_2_ Crystal	0.01 M TBAC in Acetonitrile	−3.2 V for 4–5 h (Two‐Electrode System)	≈3 nm, 50 nm to several micrometers	[90]
2D Polymeric C_60_	Bulk Mg_4_C_60_ Crystals	TBA·HSO_4_	−8 V for 2 h (Two‐Electrode System)	≈2.1 nm (Average), ≈10 um	[127]

### 3.3 Electro‐Topotactic‐Transformation of 2D Materials

**Figure 7:**
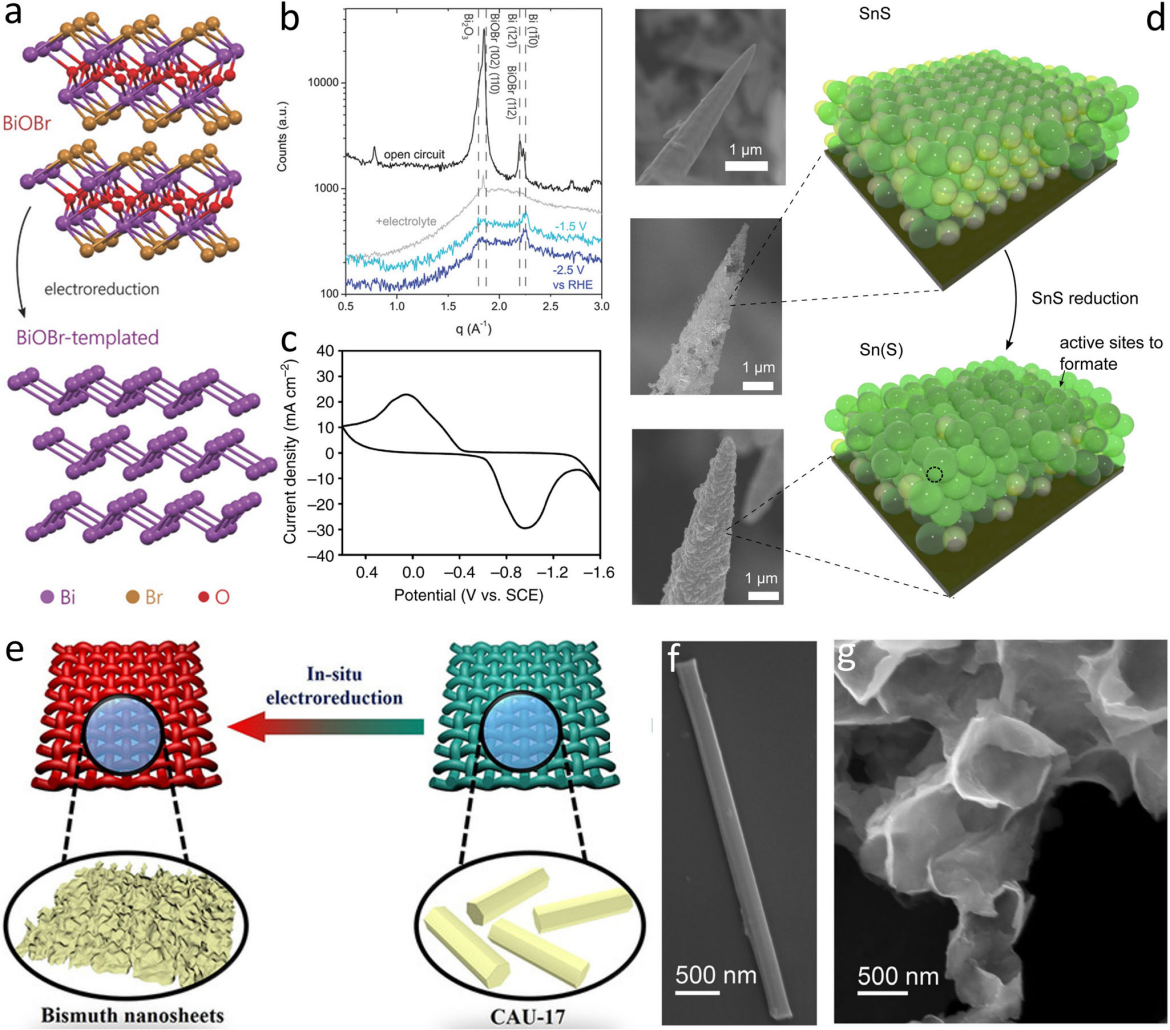
Electrochemical topotactic transformation for the synthesis of 2D materials. a) Electrochemical topotactic transformation of 2D bismuth oxyhalides to 2D Bi catalysts. b) In operando GIWAXS measurements elucidating the structural evolution of 2D metal oxyhalide‐derived catalysts at open‐circuit potential and in the absence of electrolyte. Reproduced with permission.^[^ ^92^ ^]^ Copyright 2018, Wiley‐VCH. c) CV curve of precursor BiOI nanosheets in NaHCO_3_ electrolyte. Reproduced with permission.^[^ ^38^ ^]^ Copyright 2020, Springer Nature. d) Electrochemical production of sulfur‐modulated Sn nanostructured catalysts on Au needles via partial reduction. Reproduced with permission.^[^ ^94^ ^]^ Copyright 2017, Cell Press. e) In situ electrochemical reconstruction and reduction of Bi‐based MOF nanorods to 2D Bi nanosheets. Reproduced with permission.^[^ ^57^ ^]^ Copyright 2021, Wiley‐VCH. SEM images of f) initial Bi‐MOF nanorods and g) electrochemically reconstructed Bi nanosheets. Reproduced with permission.^[^ ^58^ ^]^ Copyright 2021, Wiley‐VCH.

2D Bi‐based layered materials, including BiOX (Cl, Br, and I), Bi_2_O_2_CO_3_, and Bi_2_Mo/WO_6_, can be electrochemically topotactical transformed into 2D ultrathin Bi nanosheets without significant structural deformation (**Figure** **7**a).^[^ ^91^, ^92^, ^93^ ^]^ The structural evolution of [Bi_2_O_2_]‐templated compounds in electroreduction processes was investigated via in operando grazing‐incidence wide‐angle X‐ray scattering (GIWAXS) measurements (Figure 7b), revealing that Bi nanolayers with preferentially exposed facets can in situ formed via electrochemical topotactic reduction of Bi‐based templates in electrocatalytic reactions.^[^ ^92^ ^]^ Moreover, the electrochemical reduction process of Bi‐based metal oxyhalide to metallic Bi at cathodic potentials was also confirmed by the cyclic voltammetry (CV) curve in 0.5 M NaHCO_3_ electrolyte (Figure 7c), which exhibited a pair of nearly symmetric redox waves between –1.4–0.5 V versus saturated calomel electrode (SCE) ascribed to the reversible interconversion between Bi^3+^ and metallic Bi.^[^ ^38^ ^]^ In addition, the partial electro‐reduction of precursors can be utilized for the synthesis of surface‐modified 2D materials. For example, most of the sulfur atoms can be removed from SnS_2_ during the electrochemical reduction step, finally generating S‐modulated Sn catalysts (Figure 7d).^[^ ^94^ ^]^ The morphological reconstruction of 2D materials can be achieved under the reduction potentials (Figure 7e).^[^ ^57^ ^]^ In the electrochemical reduction process, Bi(1,3,5‐tris(4‐carboxyphenyl)benzene) MOF underwent a structural change from Bi‐based MOF nanorods (Figure 7g) to 2D Bi nanosheets (Figure 7f).^[^ ^58^ ^]^ Additionally, atomically thick 2D non‐noble‐metal alloy (e.g., NiFe, NiCo, CoFe, and NiCoFe) nanosheets can be also prepared by the electrochemical topotactic reduction of ultrathin layered double hydroxides (LDH) precursors.^[^ ^95^ ^]^ This synthetic approach can not only effectively retain the initial morphology of the precursors, but also keep the atomic dispersion of their metallic compositions (**Table** **5**).

**Table 5:** Electrochemical topotactic transformation for synthesis of 2D materials.

Product	Precursor	Electrolyte	Electrochemical engineering parameters	Dimensions (Thickness, size)	Refs.
Bi_2_O_2_(CO_3_)_x_Cl_y_	BiOCl Nanosheets	CO_2_‐Saturated 0.5 M KHCO_3_	−0.8 V vs RHE for 2h	130‐140 nm, 600–800 nm	[35]
2D Bi Nanosheets	BiOI Nanosheets	0.5 M NaHCO_3_	−1.55 V vs SCE for 2 h	**/**	[38]
2D Bi Nanosheets	Bi‐based MOF	CO_2_‐Staturated 0.5 M KHCO_3_	−1.6 V vs Ag/AgCl for 2 h	12 nm, 2–10 um	[57]
2D Bi Nanosheets	Ultrathin 2D‐BOON Nanosheets	CO_2_ Saturated 1 M KHCO_3_	50 mV s^−1^ for 10 cycles, ‐0.6 V to ‐2 V vs Ag/AgCl	(2D petal‐like layers)	[92]
2D Bi Nanosheets	BiOCl Nanosheets	Ar‐Saturated 0.5 M KHCO_3_	50 mV s^−1^ for 100 cycles, ‐0.6 to ‐1.4 V vs Ag/AgCl	**/**	[93]
S‐Modulated Sn Nanosheets	SnS_x_ Thin Films	CO_2_‐Saturated 0.1 M KHCO_3_	50 mV s^−1^ for 3 cycles, 0.4 V to ‐1.0 V vs RHE	2–3 nm (Thickness)	[94]
2D Bi Nanosheets	Bismuth Nitrate Nanosheets	CO_2_‐Saturated 0.5 M KHCO_3_	−1.6 V vs SCE for 10 min	**/**	[120]

### 3.4 Electro‐Deposition for Preparation and Modification of 2D Materials

Various 2D nanoarchitectures, such as atomically thin 2D heterostructures and single‐atom metals anchored on 2D layered materials, can be rationally designed, and constructed using in situ electrochemical deposition method (**Table** **6**).^[^ ^96^ ^]^

**Table 6:** Electrochemical deposition strategy for production or modification of 2D materials.

Product	Substrate	Electrolyte	Electrochemical engineering parameters	Dimensions (Thickness, size)	Refs.
Large‐Area MoS_2_	Graphene	0.1 M (NH_4_)_2_S_2_O_8_	**/**	7‐8 Layers (Thickness)	[100]
Ir SA/MoS_2_	MoS_2_ Nanosheets	100 µM Metal Chlorides	5 mV s^−1^, 0.10 V to −0.40 V (cathodic deposition), or 1.10 V to 1.80 V (Anodic Deposition)	**/**	[106]
Pt SA/NiFe‐LDH	NiFe‐LDH Nanosheets	1 M KOH	5 mVs^−1^, 0.05 to ‐0.50 vs RHE for 40 Cycles	**/**	[107]
Pt Nanoparticles/ 2H‐WS_2_ Nanosheets	2H‐WS_2_ Nanosheets	0.5 M H_2_SO_4_	50 mV s^−1^, 0 V to ‐0.5 V vs RHE	**/**	[119]
MoS_2_ Thin Films	FTO Glass	2 mM (NH_4_)_2_S_2_O_8_	10 mV s^−1^, ‐1.2 V to ‐0.25 V vs SCE	≈250 nm (Thickness of Film)	[124]
M (Pt, Au, Pd) SA/MoS_2_	2H‐MoS_2_ Nanoflakes	0.5 M H_2_SO_4_	1.9‐2.3 V vs RHE for 1–24 h	≈15 nm MoS_2_ Film (Thickness)	[125]

**Figure 8:**
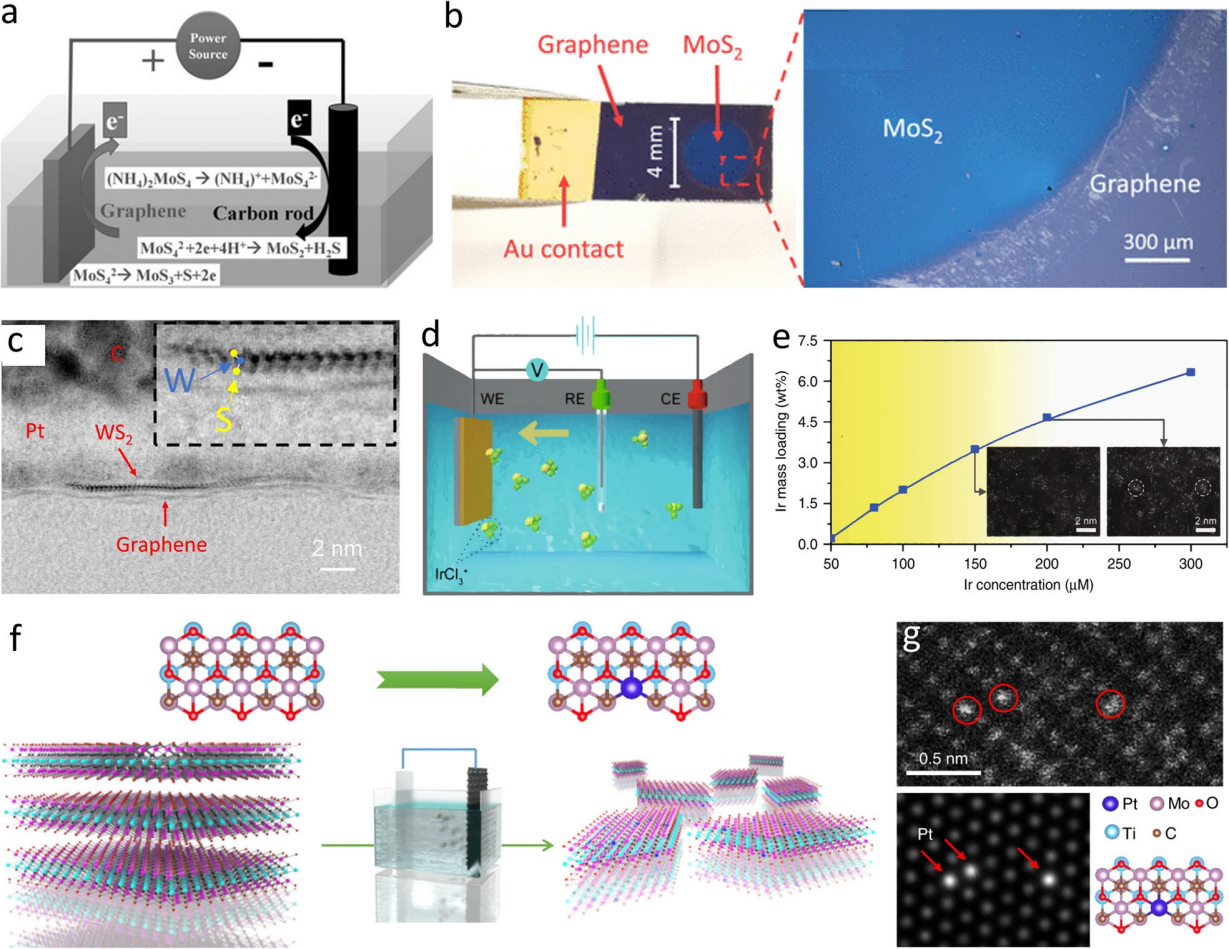
Electrochemical deposition for construction of 2D architectures. a) Schematic diagram of the controlled electrochemical deposition for the fabrication of high‐quality, large‐area, and low‐cost MoS_2_/graphene vertical heterostructures. Reproduced with permission.^[^ ^100^ ^]^ Copyright 2017, Wiley‐VCH. b) Photograph of large‐area MoS_2_/graphene heterostructure electrodeposited on Si/SiO_2_ substrate. Reproduced with permission.^[^ ^101^ ^]^ Copyright 2020, American Chemical Society. c) Atomic‐resolution TEM image of monolayer WS_2_ electrodeposited over graphene electrodes. Reproduced with permission.^[^ ^102^ ^]^ Copyright 2022, IPO Publishing. d) Schematic of the electrochemical cathodic deposition of noble‐metal species on 2D supports. e) Mass loadings of single atoms on supports as a function of the corresponding metal concentration in the electrolyte. Reproduced with permission.^[^ ^106^ ^]^ Copyright 2020, Springer Nature. f) Schematic of the electrochemical deposition process of single Pt atoms onto exfoliated MXene nanosheets. g) Atomic resolution HAADF‐STEM image of isolated Pt atoms on Mo_2_TiC_2_T_x_ nanosheets and its corresponding simulated image. Reproduced with permission.^[^ ^39^ ^]^ Copyright 2018, Springer Nature.

Atomically thin heterostructures of 2D materials with unexpected electronic and chemical properties have drawn intense scientific interest, especially in electronic and optoelectronic applications.^[^ ^97^, ^98^, ^99^ ^]^ In electrochemical deposition processes, the controllable diffusion of ions in varying concentrations and the reaction solvents can significantly release the lattice strain due to lattice mismatches between counterparts, which will be favorable for heteroepitaxial growth of 2D heterostructures. Taking 2D MoS_2_/graphene vertical heterostructure as an example, the wafer‐scale continuous MoS_2_ film on graphene‐based substrates can be produced via the electrochemical deposition approach at low costs with graphene as the anode, carbon rod or another graphene as the cathode, and ammonium tetrathiomolybdate (NH_4_)_2_MoS_4_ as the electrolyte (**Figure** **8**a).^[^ ^100^ ^]^ At the anode electrode, molybdenum trisulphide (MoS_3_) thin‐film uniformly grows on top of monolayer graphene sheet in electrochemical deposition process via the oxidative reaction: MoS_4_^2‐ –^ 2e^−^ → MoS_3_ + S. Meanwhile, a two‐electron reduction reaction occurs at the cathode electrode: MoS_4_^2−^ + 2e^−^ + 4H^+^ → MoS_2_ + 2H_2_S. Then, after annealing treatment of the as‐fabricated samples under a protective atmosphere, 2D vertical heterostructures composed of large‐area few‐layer MoS_2_ film on the graphene‐based substrate can be obtained. Several factors related to electrochemical deposition processes, such as current density, deposition time, electrolyte type and concentration, have a significant impact on the properties of electrodeposited 2D materials. For example, the thickness of deposited MoS_2_ nanolayers can be effectively tuned by controlling the deposition time. To overcome drawbacks of the two‐electrode aqueous electrodeposition, Noori et al. demonstrated nonaqueous electrodeposition using a three‐electrode electrochemical setup to successfully grow large‐scale ultrathin MoS_2_ film on graphene electrode (Figure 8b).^[^ ^101^ ^]^ Moreover, other TMDs atomic layers, such as WS_2_ monolayers, can also be site‐selectively deposited on graphene electrodes to construct 2D heterostructures using a single‐source precursor (Figure 8c).^[^ ^102^ ^]^

The incorporation of metal single‐atom dopants into 2D nanomaterials can not only introduce additional catalytically active sites, but also optimize the adsorption interactions between the active sites and reaction intermediates during catalytic processes.^[^ ^103^, ^104^ ^]^ However, general synthetic strategies for the fabrication of single atoms supported on 2D materials often have special requirements for the isolated metal atoms and 2D nano‐supports.^[^ ^105^ ^]^ To overcome these limits, the electrochemical deposition as a general synthetic route for introducing heteroatoms onto 2D supports and precisely tailoring their coordination environments and valence states was reported, which is applicable to a wide range of metals anions (e.g., Ru, Rh, Pd, Ag, Pt, and Au) and 2D supports (e.g., Co(OH)_2_, MoS_2_, and Co_0.8_Fe_0.2_Se_2_) (Figure 8d).^[^ ^106^ ^]^ During the electrochemical synthesis, the cations were transferred toward the cathode by the applied electric field and were electrochemically deposited on the supports.^[^ ^107^ ^]^ Taking Ir electrodeposition as an example, the mass loadings of Ir species constantly rose with the increase of Ir concentration and high mass loading up to 4.7 wt.% of Ir single atoms on 2D nanosheets can be achieved (Figure 8e). In addition, a variety of high‐quality single atoms on 2D atomically thin nanolayers were proposed and developed via in situ electrochemical deposition procedures. In situ electrochemical deposition of Pt single atoms on Mo_2_TiC_2_T_x_ nanosheets were achieved by conducting repeated linear sweep voltammetry scans using a three‐electrode electrochemical setup with Pt foil as the counter electrode and Mo_2_TiC_2_T_x_ on carbon paper as the working electrode.^[^ ^39^ ^]^ During this electrochemical process, Pt dissolution occurs on the surface of Pt foil in acidic media. Then dissolved Pt species (Pt^2+^ or PtO_x_) move to the working electrode and get trapped by Mo vacancy sites and reduced to isolated Pt atoms (Figure 8f). The atomic structure of single Pt atoms immobilized on MXene nanosheets was confirmed by HAADF‐STEM (Figure 6g) and DFT simulation.

## 4 Applications

Emerging 2D nanomaterials prepared or modified by electrochemical processing approaches have been widely explored for potential applications on electrochemical energy storage and conversion, electronics/optoelectronics, etc.^[^ ^26^, ^89^, ^108^, ^109^ ^]^

### 4.1 Electrocatalytic Energy Conversion

Electrocatalytic energy conversion has been viewed as one of the most promising energy‐converting pathways realizing sustainable energy conversion and utilization in the future.^[^ ^103^, ^110^, ^111^, ^112^, ^113^, ^114^ ^]^ The performances of electrocatalysts are heavily dependent on their morphological characteristics, atomic structures, and electronic properties.^[^ ^115^, ^116^, ^117^, ^118^ ^]^ Electrochemical engineering strategies have been exploited to provide precise engineering of 2D nanolayers to boost electrocatalytic activities for HER, oxygen evolution reaction (OER), and carbon dioxide reduction reaction, etc.

**Figure 9:**
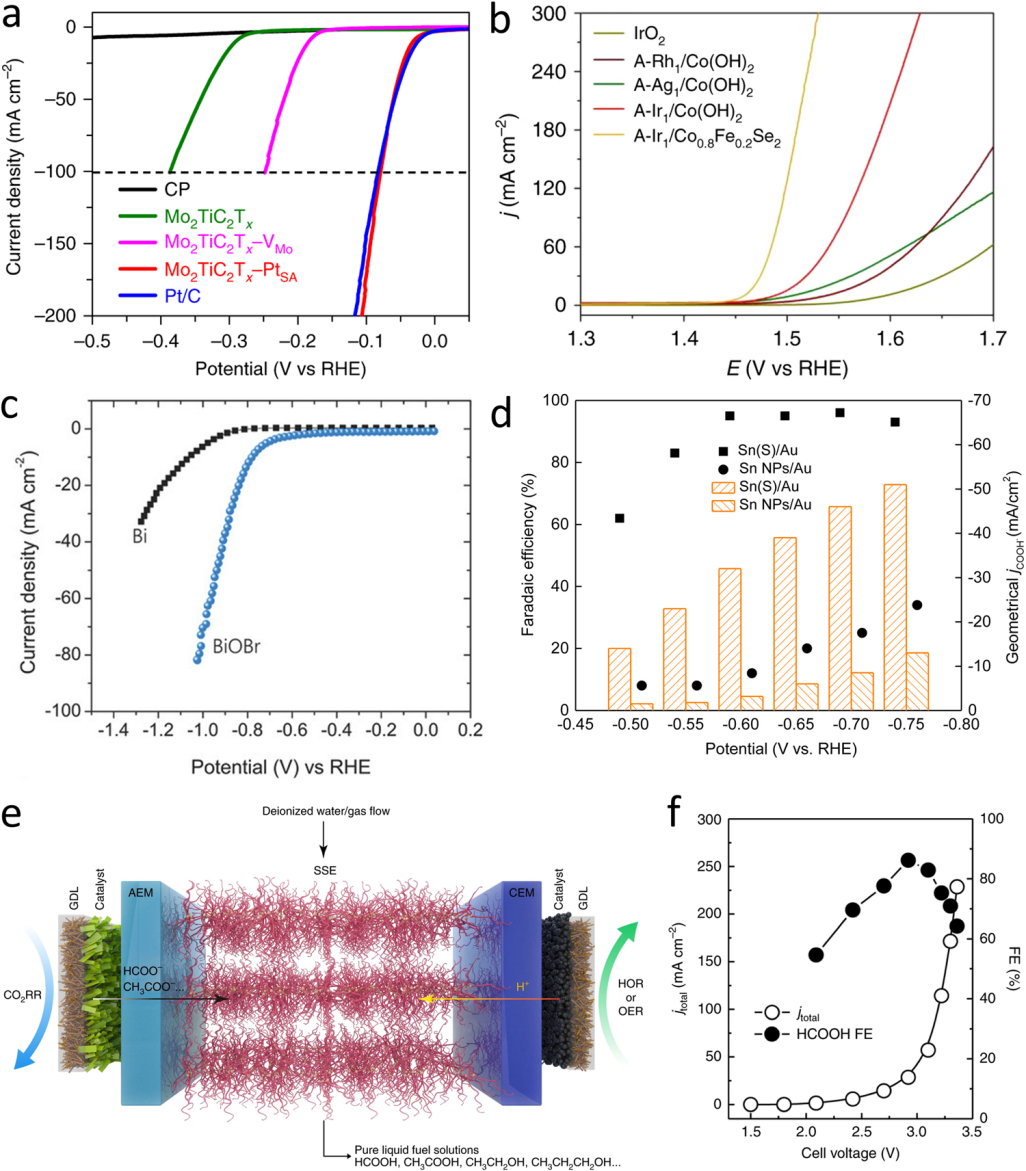
Applications of electrochemically engineered 2D materials in electrocatalytic energy conversion. a) HER polarization curves of Pt single‐atom catalysts supported on MXene fabricated by in situ electrochemical deposition process in 0.5 M H_2_SO_4_ solution. Reproduced with permission.^[^ ^39^ ^]^ Copyright 2018, Springer Nature. b) Polarization curves of noble‐metal single‐atom catalysts cathodically deposited on 2D Co‐based support for OER in 1.0 M KOH. Reproduced with permission.^[^ ^106^ ^]^ Copyright 2020, Springer Nature. c) Linear sweep voltammograms of electrochemically derived 2D Bi‐based catalysts. Reproduced with permission.^[^ ^92^ ^]^ Copyright 2018, Wiley‐VCH. d) Potential versus Faradaic efficiencies and current densities for CO_2_ reduction reaction on electrochemically reduced Sn(S) catalysts. Reproduced with permission.^[^ ^94^ ^]^ Copyright 2017, Cell Press. e) Schematic illustration of the solid‐electrolyte electrolytic cell for CO_2_ reduction reaction with an ultrathin 2D‐Bi catalyst synthesized by in situ electrochemical reduction. f) Current density‐voltage profile and the corresponding HCOOH Faradaic efficiencies of optimized CO_2_ reduction system. Reproduced with permission.^[^ ^120^ ^]^ Copyright 2021, Springer Nature.

The coordination environment of single‐atom catalysts on 2D supports can be precisely engineered by electrochemical deposition to enhance electrocatalytic activity.^[^ ^119^ ^]^ Zhang et al. demonstrated that single Pt atoms electrochemically deposited on Mo_2_TiC_2_T_x_ nanosheets were anchored via Pt‐C covalent bonds, delivering remarkable HER activity in 0.5 M H_2_SO_4_ electrolyte with low overpotentials of 30 and 77 mV to obtain 10 and 100 mA cm^−2^, respectively. In fact, the catalytic performance of optimized Pt single‐atom catalyst outperformed that of commercial Pt/C catalyst (**Figure** **9**a).^[^ ^39^ ^]^ For electrocatalytic OER, newly explored single‐atom catalysts confined on 2D materials, such as Rh_1_/Co(OH)_2_, Ag_1_/Co(OH)_2_, Ir_1_/Co(OH)_2_, and Ir_1_/Co_0.8_Fe_0.2_Se_2_, fabricated via electrochemical anodic deposition all exhibited superior activities in 1.0 M KOH, exceeding the catalytic performance of the commercial IrO_2_ catalysts (Figure 9b).^[^ ^106^ ^]^

Ultrathin 2D Bi electrocatalysts synthesized via electroreduction of metal oxyhalide exhibited exceptional capability to electrochemically catalyze CO_2_ reduction to formate compared with thermally evaporated Bi (Figure 9c).^[^ ^92^ ^]^ The enhanced electrocatalytic performance mainly originated from preferential exposure of more active Bi facets created by the electroreduction process. For partially reduced S‐modulated Sn sample, the Faradaic efficiency of nearly 100% toward formic acid formation can be achieved for potentials lower than ‐0.6 V versus RHE, and the corresponding current density is much higher than that of Sn‐based nanoparticles (Figure 9d).^[^ ^94^ ^]^ To enhance a continuous production of liquid fuel solutions, such as HCOOH, acetic acid, ethanol with ultrahigh purity, a solid‐state electrolyte was employed for the alternative of the traditional liquid electrolyte in an electrocatalytic CO_2_ reduction reaction system (Figure 9e).^[^ ^120^ ^]^ The in situ electro‐reduced 2D‐Bi catalysts obtained via topotactical transformation of 2D layered bismuth nitrate‐Bi_6_O_6_(OH)_3_(NO_3_)_3_∙1.5H_2_O (BOON) exhibit excellent CO_2_ reduction reaction performance for the production of pure HCOOH solution with concentrations reaching up to 12 M in the rationally designed CO_2_ reduction cell with solid electrolytes (Figure 9f).

### 4.2 Electrochemical Energy Storage

**Figure 10:**
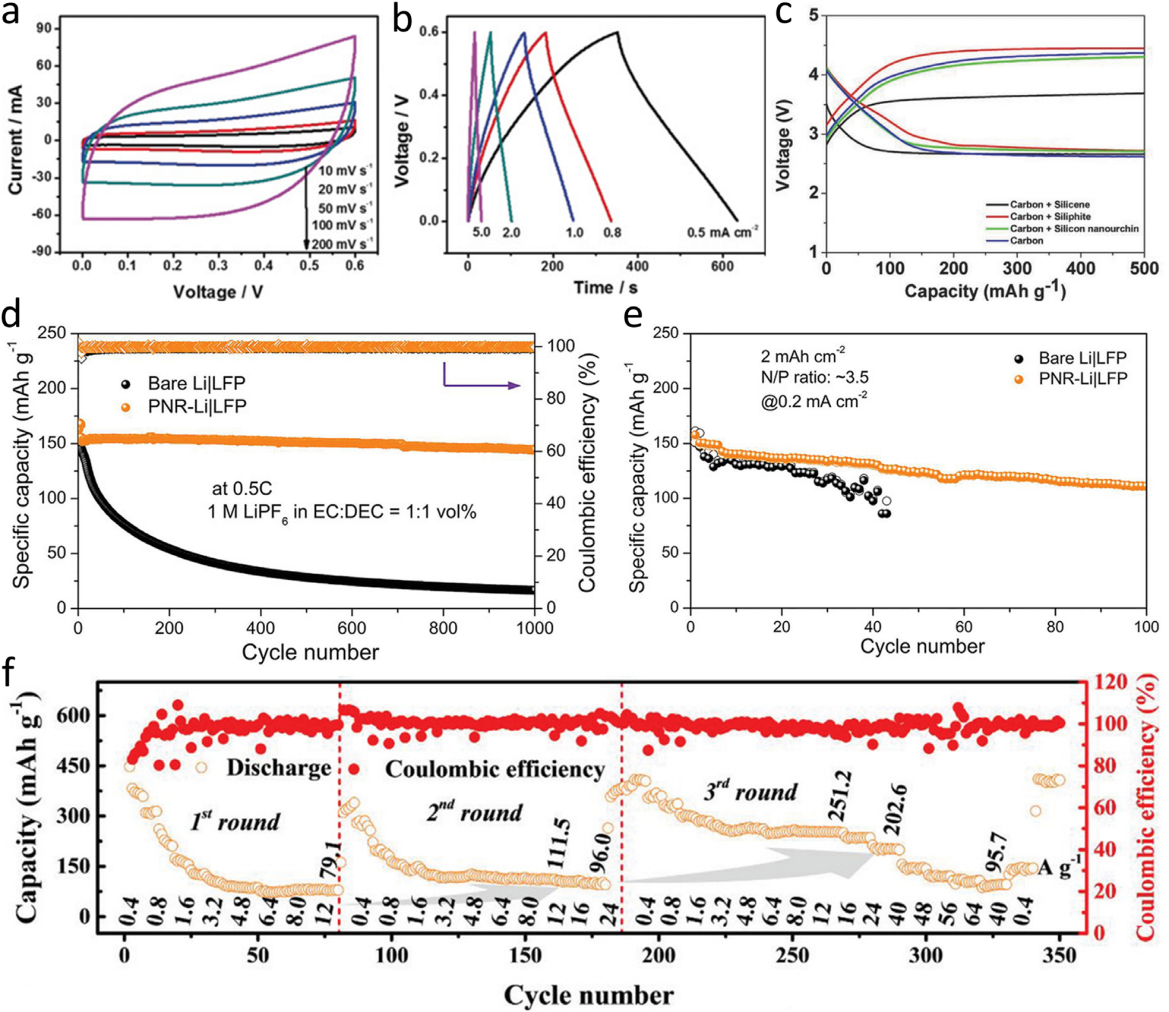
Applications of electrochemically engineered 2D materials in electrochemical energy storage. Electrochemical performances of the MXene‐based all‐solid‐state supercapacitor: a) cyclic voltammetry curves at scan rates from 10 to 200 mV s^−1^ and b) galvanostatic charge–discharge curves at current densities from 0.5 to 5 mA cm^−2^. Reproduced with permission.^[^ ^86^ ^]^ Copyright 2018, Wiley‐VCH. c) Representative charge‐discharge voltage profiles of silicene‐based electrodes used for Li‐O_2_ batteries. Reproduced with permission.^[^ ^75^ ^]^ Copyright 2018, Wiley‐VCH. Galvanostatic cycling curves of the LiFePO_4_|phosphorene‐Li full‐cell batteries: d) LiFePO_4_ as the cathode and Li metal with/without protective layers of phosphorene nanoribbons as the anode, e) LiFePO_4_ as the cathode and phosphorene‐Li or bare Li as the anode with an N/P ratio of 3.5 and an areal capacity of 2 mAh cm^−2^. Reproduced with permission.^[^ ^72^ ^]^ Copyright 2021, Wiley‐VCH. f) Rate performance of the V‐based aqueous zinc ion battery using the V_2_CT_X_ electrode for three consecutive rounds. Reproduced with permission.^[^ ^89^ ^]^ Copyright 2020, Wiley‐VCH.

Electrochemically functionalized or fabricated 2D nanolayers also have great promises for applications in electrochemical energy storage systems, including supercapacitors and rechargeable batteries (e.g., Li‐O_2_ battery, Li‐ion battery, and aqueous Zn‐ion battery).^[^ ^26^, ^29^ ^]^ Owing to the pseudocapacitive storage mechanism, MXene is a promising material for the application in supercapacitors.^[^ ^54^ ^]^ For example, the all‐solid‐state supercapacitor based on fluoride‐free electrochemically etched Ti_3_C_2_T_x_ electrode delivered a remarkable areal capacitance of 220 mF cm^−2^, outperforming those assembled with MXenes fabricated via the wet‐chemical etching approaches (**Figure** **10**a,b).^[^ ^86^ ^]^ Silicene prepared by the electrochemical lithiation/delithiation process exhibited high performance for rechargeable Li‐O_2_ battery. As displayed in Figure 10c, silicene electrode showed the lowest charging voltage among all the samples, thus resulting in a high energy efficiency of 73%.^[^ ^75^ ^]^ It was found that the silicene nanosheets existed in the allo‐Si phase, and multiple oxygen‐containing functional groups including Si(‐O)_2_, Si(‐OH)_x_, and Si(‐O)_4_ were presented on their surfaces, contributing to the outstanding performances.

Due to a remarkable abundance of active edge sites, the phosphorene nanoribbon can spontaneously react with Li metal to generate Li_3_P phase with high Li^+^ ion conductivity, acting as a protective layer on Li metal anode (Figure 10d).^[^ ^72^ ^]^ The formed protective layer can effectively hinder the parasitic reaction between the lithium metal and electrolyte and facilitate Li^+^ ion diffusion rate, resulting in the suppression of Li dendrite formation and superior cycling performance (Figure 10e). Moreover, the aqueous zinc ion battery based on the V_2_CT_X_ electrode exhibited excellent long‐time cycling stability maintaining performance for up to 1100 h at a current density of 1 mA cm^−^^2^ and rate performance achieving an areal capacity of 2 mAh cm^−2^. It was obvious that the discharge capacity at the same current density showed an increasing trend as the cycle increases, and a high discharge capacity of 95.7 mAh g^−1^ was retained at 64 A g^−1^ in the third round (Figure 10f), outperforming most existing V‐based zinc ion batteries.^[^ ^89^ ^]^

### 4.3 Electronic/Optoelectronic Devices

Besides the applications in energy conversion and storage, 2D materials engineered by electrochemical routes have also been explored and demonstrated in electronic and optoelectronic devices (e.g., solar cell, field effect transistor, photodetector, and superconducting device).^[^ ^28^, ^47^, ^109^ ^]^

**Figure 11:**
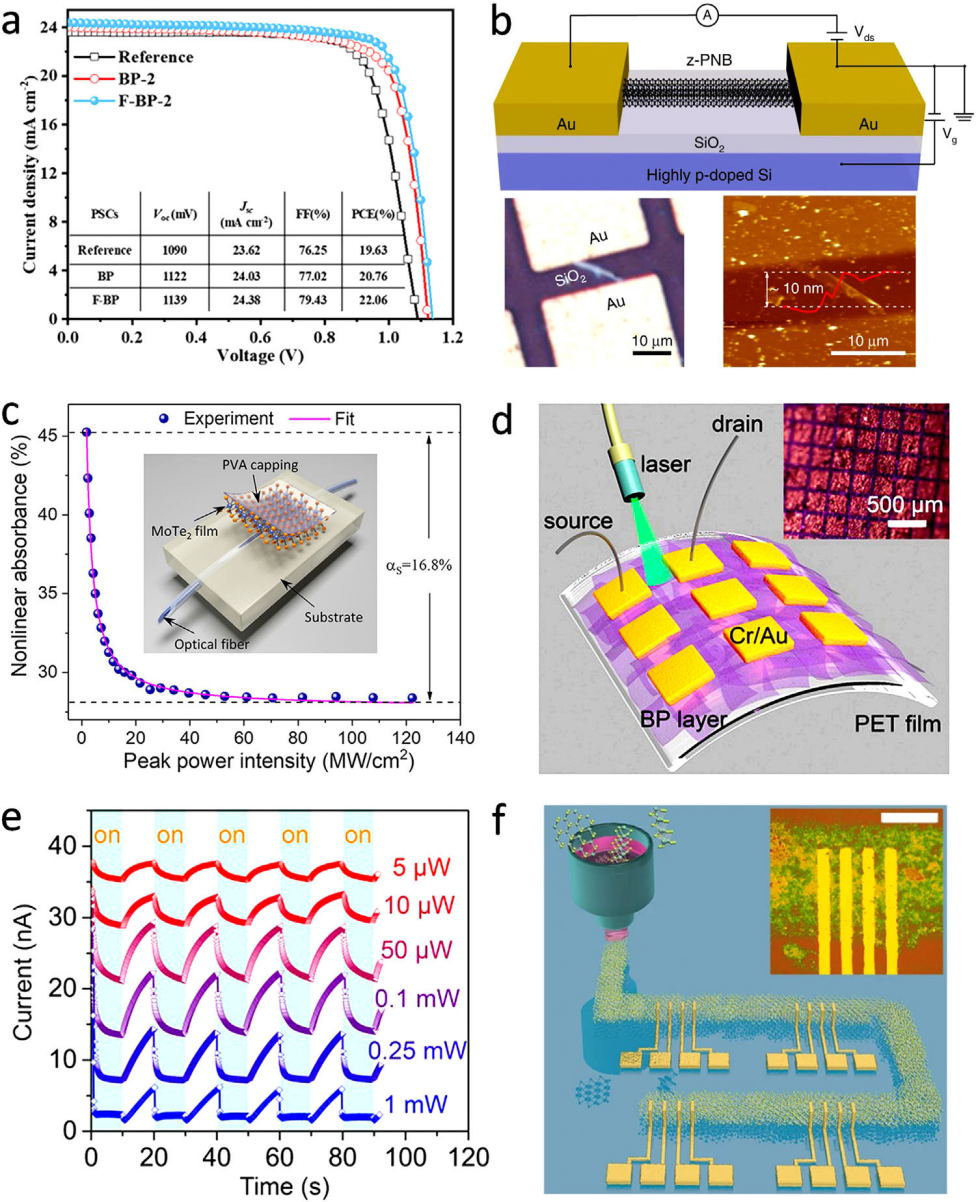
Electronic/optoelectronic applications of electrochemically engineered 2D materials. a) *J*–*V* curves of the optimised perovskite solar cells with the incorporation of BP and F‐BP nanosheets under simulated AM 1.5G solar illumination. Reproduced with permission.^[^ ^121^ ^]^ Copyright 2021, Wiley‐VCH. b) Schematic of a bottom‐gated three‐terminal device based on individual zigzag‐phosphorene nanobelt with a thickness of ≈10 nm. Reproduced with permission.^[^ ^71^ ^]^ Copyright 2020, Springer Nature. c) Nonlinear absorbance versus the input peak intensity based on an optical modulation device with 1T’‐MoTe_2_ monolayers serving as saturable absorbers. Reproduced with permission.^[^ ^76^ ^]^ Copyright 2021, American Chemical Society. d) Schematic illustration of the large‐area photodetector constructed with few‐layer phosphorene thin films. e) Photo‐response of the few‐layer phosphorene optical devices under global irradiation using a 532 nm laser with different light intensities. Reproduced with permission.^[^ ^70^ ^]^ Copyright 2018, American Chemical Society. f) Schematic illustration of a large‐area superconducting wire composed of NbSe_2_ nanoflakes patterned on SiO_2_ substrate using 3D inkjet printing. Reproduced with permission.^[^ ^81^ ^]^ Copyright 2021, Springer Nature.

Fluorinated BP (F‐BP) nanosheets synthesized by one‐step electrochemical delamination was incorporated into perovskites/HTL interlayer for successful construction of perovskite solar cells with high efficiency and long‐term stability. The formation of a strong hydrogen bond between F^−^ and MA^+^/FA^+^ as well as an ionic bond between F^−^ and Pb^2+^ at the perovskite/F‐BP interface results in the effective reduction of interfacial trap density, contributing to extremely high hole mobility and superior power conversion efficiency (22.06%) of F‐BP devices (**Figure** **11**a).^[^ ^121^ ^]^ To evaluate the electronic properties of zigzag‐phosphorene nanobelt (z‐PNB) synthesized via an electrochemical exfoliation process, a bottom‐gated three‐terminal device based on individual z‐PNB of ≈10 nm in thickness was rationally designed and constructed, which presented an ultra‐high switching ratio exceeding 10^4^ and a low leakage current of ≈10 pA meeting the requirements of active‐matrix displays (Figure 11b).^[^ ^71^ ^]^ An *n*‐type back‐gate field‐effect transistor (FET) based on the electrochemically exfoliated monolayer MoTe_2_ demonstrates a high on/off current ratio exceeding 10^6^ and field‐effect mobility of ≈1.2 cm^2^ V^−1^ s^−1^, outperforming those fabricated using mechanically, chemically, and liquid‐phase exfoliated MoTe_2_ nanosheets (Figure 11c).^[^ ^76^ ^]^ Electrochemical exfoliated few‐layer BP demonstrates excellent electronic properties and high solution processability. The associated BP‐based FET devices achieve a high mean hole mobility (≈60 cm^2^ V^−1^ s^−1^), along with an impressive average on/off ratio of ≈1 × 10^4^. Additionally, the as‐prepared BP flakes can be effectively dispersed in a broad spectrum of solvents (e.g., nonpolar, polar aprotic, and polar protic solvents). To investigate the performance of obtained ultra‐large BP nanoflakes, large‐area photodetectors were constructed by patterning Au electrode arrays on the inkjet‐printed BP films (Figure 11d).^[^ ^70^ ^]^ The illuminated experiments were carried out under global irradiation using a 532 nm laser with different light intensities (Figure 11e). Taking NbSe_2_ as a model system, a high yield (>75%) of high‐quality and large‐sized monolayer flakes (up to 300 µm) was achieved via a facile electrochemical exfoliation method.^[^ ^81^ ^]^ The functional inks of exfoliated NbSe_2_ flakes were printed and patterned on a 4‐inch SiO_2_ substrate for the fabrication of wafer‐scale 2D superconducting wire arrays using inkjet printing (Figure 11f). The obtained large‐area superconducting wire exhibits a high transition temperature (*T_c_* ≈ 6.8 K) and a high upper critical field (*H* ^⊥^_c2_ ≈ 4.4 T).

## 5 Conclusion and Perspective

Compared to traditional synthetic approaches for 2D materials, electrochemical engineering offers significant advantages in large‐scale production at low cost with simple, one‐step processing under ambient conditions. This method has been developed for manufacturing high value‐added graphene‐like 2D nanomaterials. In this review, we present four state‐of‐the‐art electrochemical approaches—exfoliation, etching, deposition, and topotactic transformation—for the synthesis and functionalization of emerging 2D materials beyond graphene. Thanks to their optimized physicochemical properties, these electrochemically engineered 2D nanomaterials show promising prospects for various applications, including electrocatalytic energy conversion and storage, electronics, and optoelectronics, etc.

Despite progress in this research area, several critical challenges remain. Future research and development in electrochemical engineering for 2D materials should focus on the following aspects: optimizing these approaches to maximize the quality and yield of 2D nanomaterials, expanding electrochemical engineering for surface modification of nanomaterials (e.g., element doping and defect engineering), and developing generalized synthetic strategies for synthesizing single atoms on 2D supports or 2D heterostructures. A thorough investigation of formation mechanisms is also essential.

Additionally, understanding the evolution of atomic and electronic structures of the precursors in electrochemical engineering processes is key to designing 2D materials with desirable properties. To investigate these fundamental processes, a wide range of advanced structural characterization technologies, such as in situ Fourier‐transform infrared spectroscopy, in situ Raman spectroscopy, and in situ X‐ray absorption spectroscopy, can be applied. The in situ electrochemical preparation of 2D materials can be further explored by integrating in situ characterization techniques, theoretical calculations, and molecular dynamics simulations. These approaches provide a more nuanced understanding of the underlying processes, enhancing insights into material synthesis and transformation mechanisms.

To fully exploit the potential of established electrochemical technologies for engineering emerging 2D materials for industrial applications, the current electrochemical production systems should be modified to enhance universality and cost‐effectiveness. Tailoring reactor components can efficiently improve the production performance of electrochemical setups. For industrial applications, dual‐electrode electrochemical cells are more suitable for commercial‐scale production due to their relatively simple structure. To lower the cost of electrochemical reactors, surface‐modified graphite‐based electrodes with high chemical stability and a wide potential window can be used to replace boron‐doped diamond or noble metal electrodes. Furthermore, to avoid laboratory safety or health issues, less hazardous intercalants or etchants should be explored as substitutes for those with high toxicity and corrosion.

## Conflict of Interest

The authors declare no conflict of interest.
